# IDAP: an integrated literature- and knowledge-graph-driven evidence prioritization pipeline for precision oncology

**DOI:** 10.1093/bioinformatics/btag300

**Published:** 2026-05-09

**Authors:** Yebin Ryu, Ha-Eun Jeong, Joon-Yong An

**Affiliations:** Department of Integrated Biomedical and Life Science, Korea University, Seoul, 02841, Republic of Korea; L-HOPE Program for Community-Based Total Learning Health Systems, Korea University, Seoul, 02841, Republic of Korea; Department of Integrated Biomedical and Life Science, Korea University, Seoul, 02841, Republic of Korea; National Research Laboratory for Convergence Degradation Biology, Korea University, Seoul, 02841, Republic of Korea; Department of Integrated Biomedical and Life Science, Korea University, Seoul, 02841, Republic of Korea; L-HOPE Program for Community-Based Total Learning Health Systems, Korea University, Seoul, 02841, Republic of Korea; National Research Laboratory for Convergence Degradation Biology, Korea University, Seoul, 02841, Republic of Korea; School of Biosystem and Biomedical Science, College of Health Science, Korea University, Seoul, 02841, Republic of Korea

## Abstract

**Motivation:**

Advances in tumor sequencing enable routine detection of dozens to hundreds of somatic alterations per patient, yet only a minority can be linked to established therapeutic evidence. Curated resources such as OncoKB provide high-quality variant-drug annotations but remain limited in coverage, particularly for rare or low-frequency variants. This coverage gap motivates computational frameworks that can integrate curated, literature-derived, graph-based, and clinical-trial evidence to prioritize therapeutic hypotheses for expert review.

**Results:**

We developed the Integrated Drug Annotation Pipeline (IDAP), a modular framework that combines four complementary evidence streams: curated variant-drug associations from OncoKB, literature-derived gene-drug mention counts from PubMed abstracts, graph-based drug prioritization using a TxGNN-derived biomedical knowledge graph, and cancer-specific clinical-trial evidence from ClinicalTrials.gov. Given a cancer type and a MAF file, IDAP generates patient-level reports summarizing detected variants, ranked therapeutic hypotheses, supporting evidence layers, and relevant clinical trials. Evaluated across five cancer types (*n = *50 samples), IDAP expanded evidence-linked therapeutic hypotheses beyond curated databases alone. Among patients without OncoKB recommendations (26/50), IDAP identified a median of 87 candidate drugs (range: 2–473). To reduce cross-source scale imbalance, the final ranking used within-sample percentile normalization with fixed bonuses for curated evidence, multi-source support, and trial linkage. Under this revised ranking, 24/50 top-ranked candidates were supported by at least two evidence sources and 44/50 had associated ClinicalTrials metadata. In an exploratory external CIViC comparison, IDAP recovered at least one matched CIViC-supported therapy in 28/41 eligible samples, with 13/41 appearing within the top 10 candidates. These outputs are intended to support evidence triage and translational interpretation rather than direct treatment recommendation.

**Availability and implementation:**

IDAP is freely available at https://github.com/joonan-lab/IDAP-pipeline, with full documentation at https://joonan-lab.github.io/IDAP-pipeline. An archived snapshot of the code used in this study is deposited on Zenodo (DOI: https://doi.org/10.5281/zenodo.19301367)

## 1 Introduction

Over the past decade, advances in genomic profiling have transformed tumor sequencing into a routine component of clinical oncology (Roychowdhury and Chinnaiyan 2014). Early approaches relied on hotspot panels or single-biomarker assays, whereas current practice increasingly employs large-scale targeted gene panels, whole-exome sequencing, and even whole-genome sequencing (Kopetz et al. 2019, Casolino et al. 2024). As a result, dozens to hundreds of somatic variants are now detected for an individual patient. Despite the promise of precision oncology, only a minority of these alterations are clinically actionable, with most representing variants of unknown significance or passenger events lacking therapeutic relevance. For example, analyses from The Cancer Genome Atlas (TCGA) (Cancer Genome Atlas Research Network et al. 2013) have shown that although approximately 40% of tumors harbor potentially actionable variants, only 7–10% can be directly matched to an FDA-approved therapy (Tsimberidou et al. 2012, Jardim et al. 2015, Zehir et al. 2017, Hoadley et al. 2018). The challenge is even more pronounced in rare cancers, where limited clinical evidence and sparse coverage in existing resources hinder the interpretation of uncommon variants.

Several clinical knowledge bases—such as OncoKB (Wagner et al. 2020) or CIViC (Krysiak et al. 2023)—provide high-quality, expert-curated variant–drug associations with defined evidence levels. However, their coverage is strongly biased toward common cancer types and recurrent driver mutations, leaving the vast majority of observed variants unannotated. Harmonized analyses across multiple knowledge bases indicate that most variants appear in only a single resource, with fewer than 10% shared across databases, highlighting substantial fragmentation of available evidence. Meanwhile, graph neural network (GNN)–based approaches such as TxGNN (Huang et al. 2024) have emerged as powerful tools for drug repurposing at the disease level and show particular promise for rare diseases. Nonetheless, these models are not variant-specific, limiting their direct applicability to precision oncology, and their black-box nature presents additional barriers to clinical adoption. Furthermore, both curated knowledge bases and machine-learning models are slow to incorporate newly published findings, despite the rapid growth of biomedical literature.

To address these limitations, we developed the Integrated Drug Annotation Pipeline (IDAP), a multi-source evidence integration framework for precision oncology. IDAP combines reliable variant-level annotations from OncoKB with graph-based drug prioritization using a TxGNN-derived biomedical knowledge graph, PubMed-derived gene-drug mention evidence, and cancer-specific clinical-trial information from ClinicalTrials.gov. Rather than replacing expert interpretation, IDAP is designed to aggregate dispersed evidence, prioritize potentially relevant therapeutic hypotheses, and generate structured reports for downstream review. By integrating curated, graph-derived, literature-derived, and trial-linked information, the framework aims to improve evidence coverage and transparency for variants and cancer contexts that are incompletely represented in existing expert-curated resources.

## 2 Methods

### 2.1 OncoKB-based variant annotation

Somatic mutation data were annotated using the official OncoKB MAF Annotator tool to obtain clinically actionable and drug-related information for each variant (Chakravarty et al. 2017). To access the tool, an API token was requested through the OncoKB API Access portal (https://www.oncokb.org/api-access), and approval was granted for research use. During the annotation process, the annotator retrieved oncogenicity classifications, clinical levels of evidence, and drug associations directly from the OncoKB database via its application programming interface (API). The input MAF files were processed under the GRCh38 reference genome, and a designated cancer type was specified so that the annotation would reflect disease-specific therapeutic evidence levels. For each variant, the annotation output included multiple fields such as ONCOGENIC, HIGHEST_LEVEL, VARIANT_IN_ONCOKB, and DrugBank identifiers, which together describe the clinical relevance and potential drug sensitivity of the mutation.

### 2.2 Literature-based drug association mapping

A literature-based mining pipeline was established using publicly available biomedical data sources. First, a comprehensive reference list of anticancer drugs was curated from the ChEMBL (Zdrazil et al. 2024) database through multiple information channels, including drug indications, pharmacological mechanisms of action, ATC classifications, and approval status. Drug names retrieved from these distinct endpoints were normalized, merged, and deduplicated to produce an extensive, unified vocabulary of clinically and experimentally relevant anticancer compounds. This standardized drug lexicon served as the foundation for downstream text-mining analyses.

Using this curated resource, we systematically queried PubMed  (Sayers et al. 2025) to retrieve abstracts relevant to the input cancer type and altered genes. For each gene, queries used the form “<cancer type> AND <gene> AND (therapy OR treatment OR inhibitor).” Retrieved abstracts were parsed, and drug mentions were identified by direct matching against the curated ChEMBL-derived drug list. The resulting associations were summarized as gene-drug mention counts, providing a literature-derived evidence layer for candidate prioritization. In the audited implementation, article-level identifiers are not retained in the final merged output table. Because abstract-level co-mentions do not by themselves establish therapeutic relevance, this module should be interpreted as evidence retrieval rather than causal inference.

### 2.3 Drug repurposing candidate discovery using knowledge graphs

We implemented a computational drug repurposing pipeline based on the TxGNN biomedical knowledge graph, which integrates 17 079 diseases, 7957 drugs, and 27 610 genes/proteins with their documented relationships. For a given cancer type and patient-specific mutated genes (e.g., EGFR, TP53, KRAS in lung cancer), the system identifies drug candidates through the following steps: (1) extraction of drugs connected to the cancer type via ‘indication’ edges (current treatments), (2) identification of drugs targeting the mutated genes via ‘drug-protein’ edges, and (3) cross-referencing with FDA-approved drugs (*n = *2055) to detect candidates approved for non-cancer or other cancer indications.

Drug candidates from the TxGNN-derived knowledge graph were prioritized using mutation-aware heuristic rules based on indication status, FDA approval status, and mutation-target relationships. In the current implementation, repurposing-priority drugs approved for other diseases and linked to altered genes received the highest scores, with additional bonuses for targeting multiple mutated genes. Drugs targeting altered genes without prior FDA approval, current-indication drugs, and broader disease-related drugs were assigned lower scores according to their evidence category. Thus, the patient-specific mutation set directly influences both candidate retrieval and ranking, while the implementation remains an interpretable graph-based prioritization procedure rather than variant-specific deep-learning inference.

### 2.4 Clinical trial–based evidence mining from ClinicalTrials.gov

Clinical trial information for candidate drugs was retrieved programmatically from the ClinicalTrials.gov registry using its v2 REST API (https://clinicaltrials.gov/api/v2/studies) (Zarin et al. 2011). For each drug in the curated anticancer drug list and a user-specified cancer type, we submitted search queries of the form “<drug name> AND <cancer type>” and requested up to 200 records per drug without additional restrictions on study phase or recruitment status. The API response was parsed to extract the National Clinical Trial (NCT) identifier, brief study title, reported disease conditions, trial phase, and overall recruitment status from the protocolSection modules. These elements were stored in a tabular format with one row per clinical trial and the following fields: drug, nct_id, title, condition, phase, and status. A short delay was introduced between consecutive queries for different drugs to avoid overloading the public API service.

To summarize the clinical development landscape for each drug, we computed a per-drug trial summary table. All trials associated with a given drug were grouped, and the number of unique NCT identifiers (n_clinical_trials) was counted. A representative “top” trial was then selected by assigning priority scores to phases (Phase 4 > Phase 3 > Phase 2 > Phase 1) and choosing one trial with the highest available phase for that drug. For each drug, the summary table reported the total number of associated clinical trials, the NCT identifier of the highest-phase trial (top_nct_id), its recorded phase (top_phase), and its brief title (top_title).

### 2.5 Integrated Drug Annotation Pipeline (IDAP) package

The Integrated Drug Annotation Pipeline (IDAP) was implemented as a modular package combining curated knowledge, graph-based prioritization, PubMed-derived mention evidence, and clinical-trial information. As input, the pipeline requires a cancer type and a MAF file. MAF parsing was used to extract altered genes, which were then propagated through the component modules. First, variants were mapped to OncoKB to retrieve curated variant-drug associations and evidence levels. Second, altered genes were mapped onto a TxGNN-derived biomedical knowledge graph to generate graph-linked drug candidates and heuristic prioritization scores. Third, the same cancer context and altered genes were used to query PubMed, and matched abstracts were summarized as gene-drug mention counts. Finally, candidate drugs were queried against ClinicalTrials.gov to attach trial metadata. For final ranking, we revised the combined score to reduce cross-source scale imbalance by converting ′txgnn_score′, ′mention_count′, and ′oncokb_score′ into within-sample percentiles (′tx_pct′, ′pm_pct′, and ′ok_pct′). The final score was then defined as ′combined_score = 0.50 x tx_pct + 0.40 x pm_pct + 0.10 x ok_pct + 0.20 x max(0, support_count - 1) + 0.30 x I(oncokb_score > 0) + 0.05 x clinical_flag′, where ′support_count′ is the number of evidence layers supporting the drug and ′clinical_flag′ indicates the presence of matched ClinicalTrials metadata. This revised rule was introduced to keep the score transparent and interpretable while preventing sparse curated evidence from being overwhelmed by the broader raw scales of the graph and literature components; it was not trained on clinical outcomes. The resulting report is intended to support expert triage and translational interpretation rather than to function as a stand-alone clinical recommendation engine.

**Figure 1:**
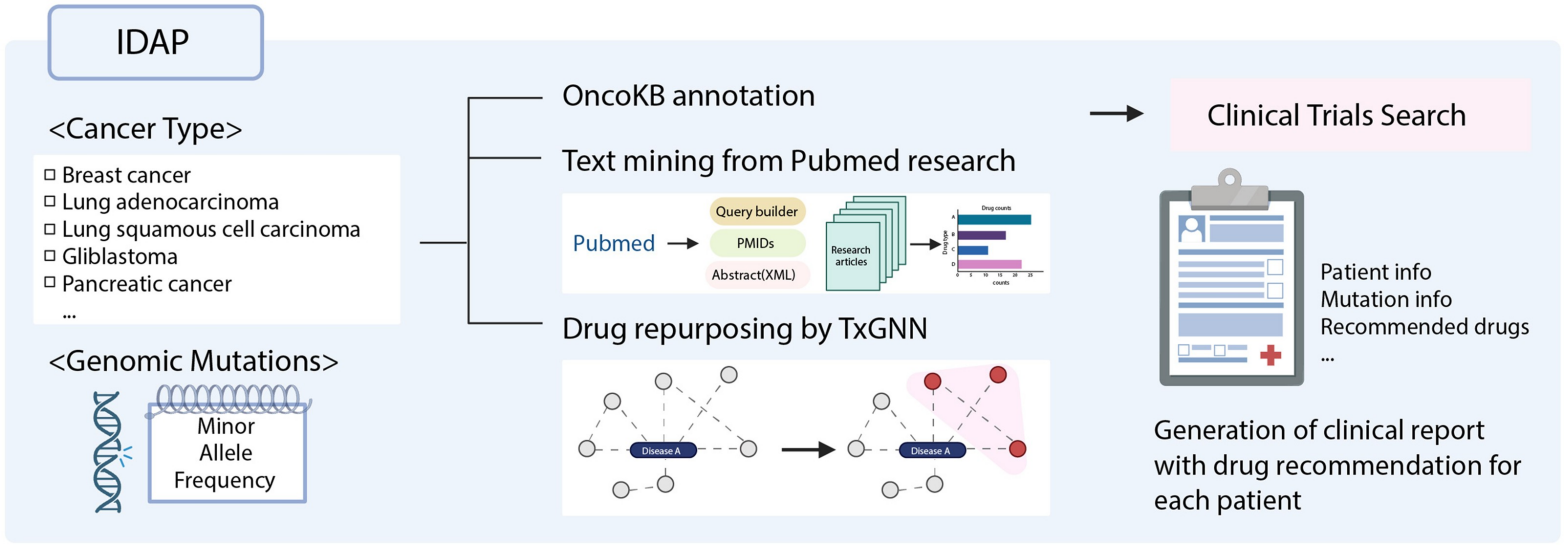
Integrated Drug Annotation Pipeline (IDAP). IDAP combines patient cancer type and genomic mutation profiles with curated annotations from OncoKB, drug repurposing predictions from TxGNN, and PubMed-derived evidence. The pipeline outputs a clinical report summarizing patient information, detected variants, and recommended drugs. Created with BioRender.com.

To evaluate the sensitivity of the ranking to the choice of percentile weights, we tested seven alternative weight configurations (Table 2, available as supplementary data at *Bioinformatics* online). The default configuration (0.50, 0.40, 0.10) was selected because it yielded the highest proportion of top-ranked candidates with multi-source support and clinical trial linkage; however, top-ranked outputs showed moderate sensitivity to weight choice, with 17/50 samples retaining the same top-1 drug across all configurations (Figure 1, available as supplementary data at *Bioinformatics* online).

### 2.6 Validation of the pipeline using public and in-house MAF files

To validate the applicability of the literature-based drug association pipeline, we tested the workflow on both internally generated and publicly available somatic mutation datasets (Song et al. 2024). Somatic variants were first called from the recalibrated BAM files using GATK4 Mutect2 (v4.1.8.0), and were subsequently annotated with GATK4 Funcotator (v4.2.0.0) to generate MAF files. In addition to these in-house MAF files, publicly available somatic mutation data from the Clinical Proteomic Tumor Analysis Consortium (CPTAC) cohorts (Li et al. 2023) were obtained via the Genomic Data Commons Data Portal (https://portal.gdc.cancer.gov).

## 3 Results

### 3.1 Construction of a unified drug-repurposing and variant-annotation framework

A unified framework for systematic and scalable annotation of cancer variants across multiple tumor types was constructed by integrating three complementary resources (Fig. 1). First, OncoKB was used to obtain curated annotations of actionable mutations together with standardized evidence levels. Second, the PubMed API was used to retrieve treatment-related literature associated with each cancer type and altered gene, and abstract-level gene-drug matches were summarized as mention counts. Third, a TxGNN-derived biomedical knowledge graph was used as a structured resource for graph-based drug prioritization, allowing the pipeline to surface drugs connected to the altered genes and cancer context through documented biological and indication relationships. These evidence streams were complemented by cancer-specific trial information retrieved from ClinicalTrials.gov.

The graph module used a TxGNN-derived biomedical knowledge graph as a structured resource for candidate discovery rather than as a variant-specific foundation-model inference engine. Drugs connected to the cancer context through indication relationships, to altered genes through drug-target relationships, or to FDA-approved repurposing opportunities were collected and then prioritized using a mutation-aware heuristic score. This design allows the graph module to surface mechanistically connected and repurposing-relevant therapeutic hypotheses in a transparent and interpretable way.

All drugs recommended through these three routes were subsequently queried on ClinicalTrials.gov using both the cancer type and candidate drug names to identify relevant ongoing or completed clinical trials. This step enabled us to link each therapeutic candidate to corresponding trial information when available.

Building on these resources, IDAP consolidates curated knowledge, literature-derived mention evidence, graph-based prioritization, and clinical-trial metadata into a single annotation workflow. The pipeline accepts a cancer type and a MAF file as input, retrieves OncoKB annotations, aggregates PubMed mention evidence, generates graph-linked candidates through the TxGNN-derived heuristic module, and attaches relevant clinical-trial information. All results are merged into an automatically generated report summarizing actionable mutations, candidate therapeutic hypotheses, supporting evidence layers, and related clinical trials across tumor types. This unified framework provides a scalable and extensible approach to variant annotation and therapeutic hypothesis prioritization for expert review.

### 3.2 Outputs generated by the IDAP

Applying IDAP to somatic mutation data yielded a structured set of patient-level outputs that summarize both variant annotations and layered drug evidence. For each case, the pipeline produced a concise overview of the cancer type, the total number of detected variants, and a ranked list of candidate therapeutic hypotheses derived from the combined score. Individual variants were linked to their corresponding clinical annotations, including oncogenicity and actionability categories, allowing users to distinguish alterations with established curated support from those supported primarily by graph-based, literature-derived, or trial-linked evidence. In a descriptive review of the ranked outputs after score revision, 24/50 top-ranked candidates were supported by at least two evidence sources and 44/50 top-ranked candidates had associated ClinicalTrials metadata; across the top five candidates per sample, 59/250 entries had support from at least two evidence layers and 195/250 had associated ClinicalTrials metadata.

At the drug level, IDAP generated a unified table in which each compound was accompanied by supporting information from curated knowledge, the biomedical literature, graph-based repurposing, and clinical trial registries. Drugs previously associated with key driver genes tended to receive high combined scores and were frequently represented among the top-ranked candidates, while additional agents with strong graph connectivity or substantial literature co-mention appeared as repurposing hypotheses. For many drugs, the pipeline also linked one or more registered clinical studies in the selected cancer type, providing direct pointers to the most advanced trial phase and representative study identifiers.

**Figure 2:**
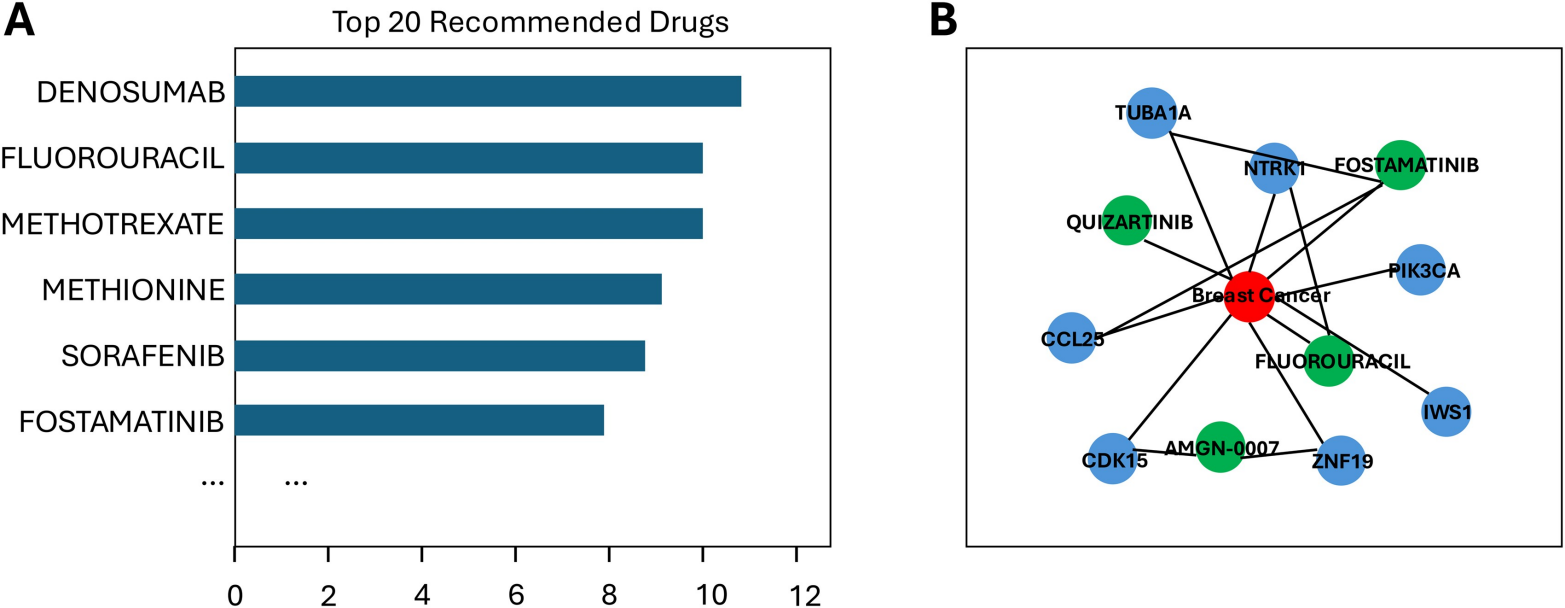
Summary of ranked therapeutic hypotheses and gene-drug relationships produced by IDAP. (A) Bar plot showing the top 20 recommended drugs ranked by the revised combined score, implemented as a sample-wise percentile-normalized integration of graph, literature, and curated evidence with fixed bonuses for multi-source support, any OncoKB evidence, and matched ClinicalTrials metadata. Higher scores indicate stronger convergent support across the implemented evidence layers. (B) Network visualization of the cancer type (red), mutated genes (blue), and recommended drugs (green). Edges represent gene-drug associations derived from the TxGNN-based graph module.

All outputs were summarized in an automatically generated report that combines tabular results with visual elements. In particular, the final document included a bar plot of the highest-scoring drugs (Fig. 2A) and a network representation of relationships among the cancer type, mutated genes, and recommended compounds, together with variant-level and drug-level tables suitable for downstream review (Fig. 2B). Taken together, these outputs illustrate how IDAP transforms raw MAF files into an interpretable overview of actionable mutations, prioritized therapeutic options, and their current level of supporting evidence.

### 3.3 Performance characteristics

**Figure 3:**
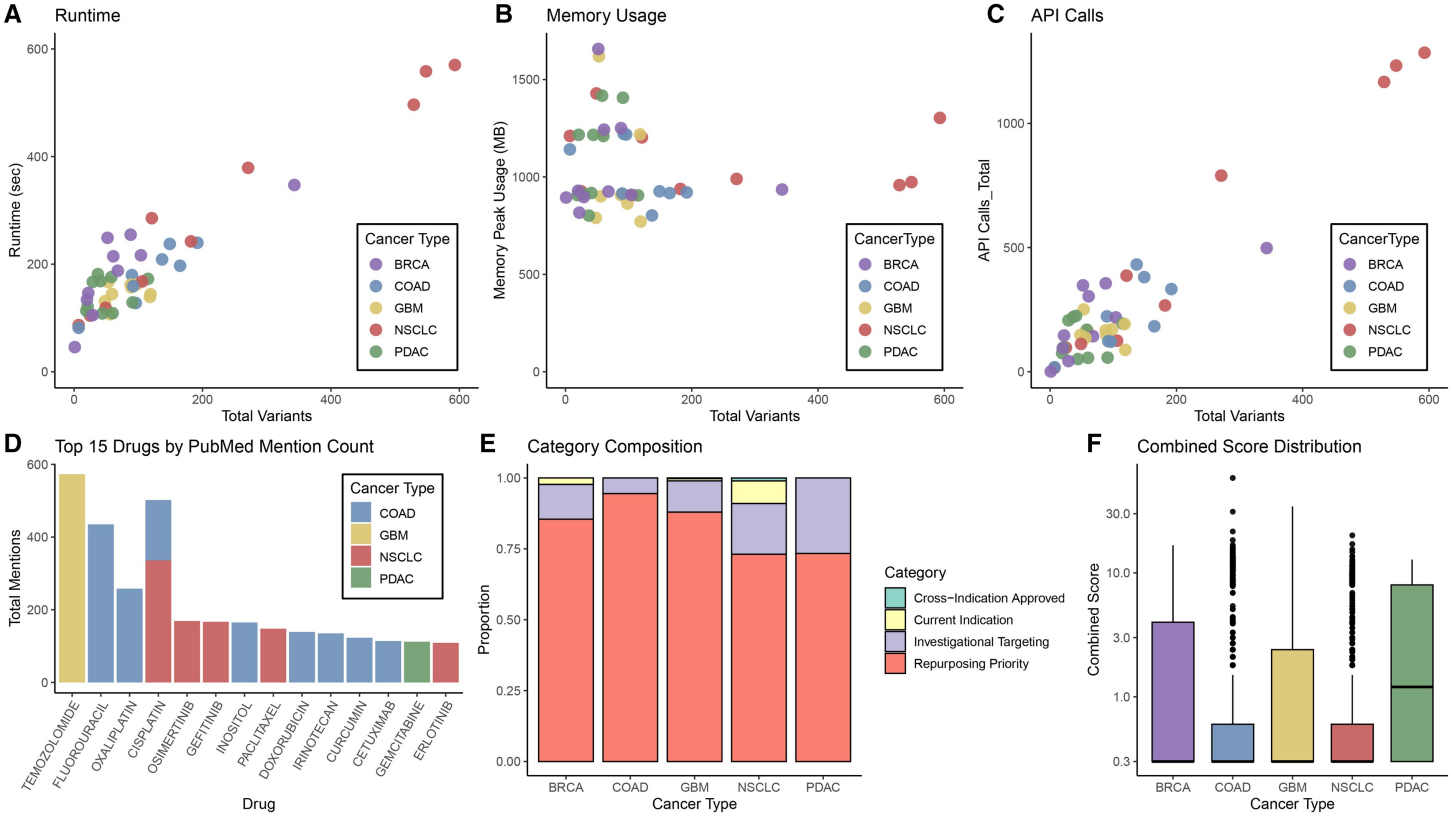
Performance and analytical characteristics of the IDAP pipeline. (A) Total runtime as a function of mutation burden across BRCA, COAD, GBM, NSCLC, and PDAC samples (*n = *10 per cancer type). (B) Peak memory usage remains consistent across samples and shows no correlation with the number of variants. (C) Total API calls per sample scale proportionally with the number of candidate drugs identified. (D) Top 15 drugs ranked by PubMed mention count, colored by cancer type of origin. Temozolomide shows dominant representation in GBM. (E) Distribution of TxGNN-derived drug categories across cancer types. Repurposing-priority drugs constitute the majority of suggestions across cohorts. (F) Combined score distribution by cancer type. PDAC exhibited higher median and broader score ranges, indicating stronger multi-source evidence support for repurposing candidates.

We evaluated the computational performance of IDAP across five cancer types (BRCA, COAD, GBM, NSCLC, PDAC; *n = *10 samples each), representing diverse mutation burdens (range: 1–3061 variants) (Table 1, available as supplementary data at *Bioinformatics* online). For clarity of visualization, three samples with exceptionally high mutation counts (>1000 variants) were excluded from performance plots. Total runtime scaled linearly with mutation burden (Fig. 3A), with a median runtime of 162 seconds (IQR: 123–221 s). Among individual modules, ClinicalTrials.gov queries accounted for the largest share of execution time (median: 84 s), followed by the TxGNN module (median: 34 s) (Table 1). Peak memory usage remained stable across all cancer types with a median of 918 MB (Fig. 3B), showing no meaningful correlation with variant count. Total API calls increased proportionally with the number of predicted candidate drugs, ranging from 1 to 3104 calls per sample (Fig. 3C).

**Table 1:** Runtime summary for each module in the IDAP workflow.

Module	Runtime summary (seconds)					
	Min	Q1	Median	Mean	Q3	Max
OncoKB	1.34	1.51	1.72	2.96	2.62	18.22
PubMed	0.73	14.14	27.22	71.50	47.59	835.72
TxGNN	41.21	61.485	69.13	64.82	74.16	81.43
ClinicalTrials	0	53.28	74.69	95.59	117.56	279.96
Total	45.78	129.44	167.46	234.87	239.21	1169.5

Beyond performance, IDAP facilitated interpretable profiling of computational outputs, including literature-derived drug salience, repurposing-related drug category distributions, and overall evidence convergence. In the PubMed analysis, temozolomide—a standard-of-care agent for GBM—was the most frequently mentioned drug across all samples (Fig. 3D). Across cancer types, most drugs exhibited strong cancer-specific literature signatures, indicating that biomedical publications remain largely focused within established disease contexts.

TxGNN-based repurposing recommendations were enriched for the repurposing-priority category, which captures drugs that are FDA-approved for other diseases and also target mutated genes present in the input tumor but are not currently indicated for the corresponding cancer (Fig. 3E). This pattern highlights the broad repurposing landscape identifiable by IDAP and its ability to surface biologically linked therapeutic hypotheses.

The combined-score distribution—reflecting integrated evidence support from PubMed, TxGNN, and OncoKB—was largely skewed toward zero across most cancer types. However, PDAC displayed noticeably higher median and overall combined scores compared to the other cohorts (Fig. 3F), reflecting a larger set of plausible repurposing candidates. Given that PDAC is historically difficult to treat, this enrichment suggests that IDAP may uncover underexplored therapeutic opportunities in hard-to-treat cancers.

### 3.4 Expanding therapeutic hypotheses for a patient with mucinous adenocarcinoma

**Figure 4:**
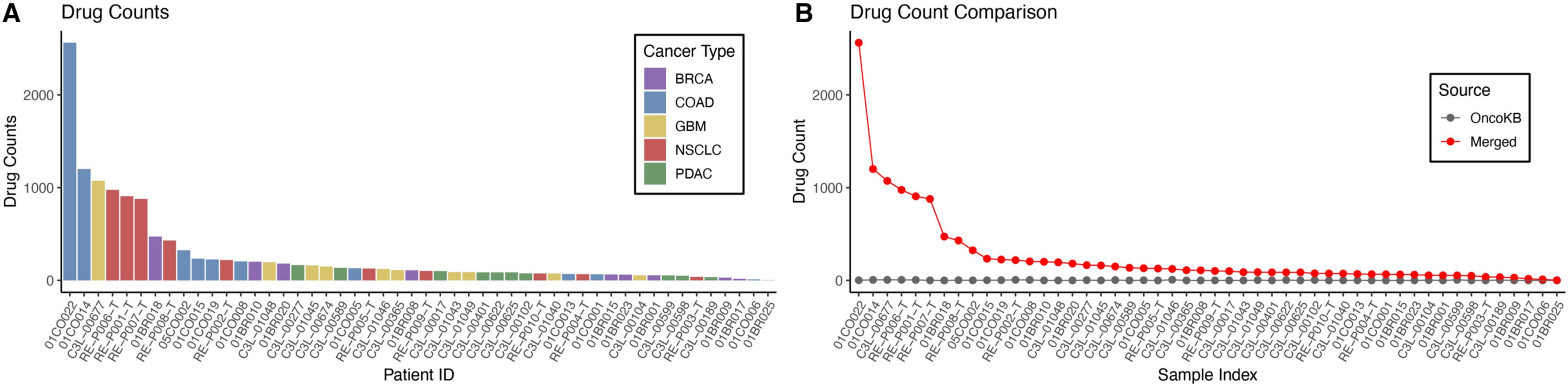
Drug annotation counts across cancer types and comparison between IDAP and OncoKB. (A) Distribution of IDAP-annotated drug counts across 50 patient samples, stratified by cancer type. (B) Comparison of drug counts generated by IDAP (red) versus conventional OncoKB annotation (gray).

The number of annotated drugs per sample varied substantially across cancer types (Fig. 4A). COAD exhibited the highest number of drug recommendations, with an average of 447 drugs per patient, followed by NSCLC, GBM, and BRCA, whereas PDAC showed the lowest average of 78 drugs per patient. Because drug count is strongly correlated with the number of variants (Fig. 3A), this distribution pattern reflects the known tumor mutational burden (TMB) characteristics of each cancer type. Consistent with previous pan-cancer studies, NSCLC and COAD are among the tumor types with the highest TMB, whereas PDAC and BRCA display relatively low mutational loads (Chalmers et al. 2017, McGrail et al. 2021).

Compared with conventional OncoKB-only annotation, IDAP produced a broader set of evidence-linked therapeutic hypotheses, particularly for cases in which curated variant-level annotations were absent. OncoKB did not return drug recommendations for 52% (26 out of 50) of patients in our dataset, whereas IDAP recovered additional ranked candidates for these cases. To better characterize the ranked outputs beyond simple count expansion, we examined evidence-layer convergence and clinical-trial linkage among the highest-ranked candidates after applying the revised score. Across the cohort, 48.0% (24/50) of top-ranked candidates and 23.6% (59/250) of top-five candidates were supported by at least two evidence sources, while 88.0% (44/50) of top-ranked candidates and 78.0% (195/250) of top-five candidates had associated ClinicalTrials metadata. We also performed an exploratory external comparison against CIViC using accepted predictive evidence items harmonized by cancer type and mutated genes. Under this benchmark, 41/50 samples had at least one gene- and disease-matched CIViC-supported therapy, and IDAP recovered at least one such therapy anywhere in the ranked list for 28/41 of these samples, compared with 6/41 for OncoKB alone. Moreover, 13/41 eligible samples contained a CIViC-supported therapy within the top 10 IDAP candidates, and the median first-match rank was 20.5. We emphasize, however, that these candidates span a range of evidence strengths and should not be interpreted as equally actionable. Instead, the combined ranking is intended to support prioritization of candidates for expert review, downstream filtering, and follow-up validation.

Analysis of the highest-scoring drugs further supported the validity of IDAP’s annotation approach. The four top-scoring drug–sample pairs were all FOSTAMATINIB in COAD and GBM samples, followed by ENDOSTATIN in NSCLC. Both drugs have documented clinical investigation histories in these malignancies. FOSTAMATINIB, an FDA-approved SYK inhibitor for immune thrombocytopenia (ITP) (Bussel et al. 2018), has been evaluated in multiple cancer types, including colorectal cancer and hematologic malignancies (Friedberg et al. 2010, Park et al. 2013). ENDOSTATIN also received high scores in NSCLC samples, aligning with its established clinical use in this indication; Endostar (recombinant human endostatin) has been approved in China for NSCLC since 2005, and combination therapy with platinum agents has shown consistently improved response outcomes in meta-analyses (Rong et al. 2012, Li et al. 2018). The concordance between IDAP’s top-ranked predictions and clinically validated therapies underscores the pipeline’s ability to prioritize biologically and clinically meaningful treatment candidates for precision oncology.

## 4 Discussion

In this study, we developed the Integrated Drug Annotation Pipeline (IDAP) as a modular evidence integration framework that consolidates curated OncoKB annotations, PubMed-derived gene-drug mention evidence, graph-based prioritization using a TxGNN-derived biomedical knowledge graph, and cancer-specific clinical-trial information from ClinicalTrials.gov. Across five cancer types, the framework expanded the number of evidence-linked therapeutic hypotheses relative to curated annotation alone, particularly for cases lacking OncoKB-supported drug matches. Rather than functioning as a clinically validated recommendation engine, IDAP is intended to provide a structured, evidence-layered report that can support translational interpretation and expert triage of therapeutic hypotheses.

Several strengths of the IDAP framework were highlighted in our analyses. First, the graph module incorporates the patient-specific mutation set directly into both candidate retrieval and heuristic prioritization, allowing drugs that target one or more altered genes to be preferentially surfaced within the broader cancer context. Although this is not model-level TxGNN reweighting, it provides an interpretable mutation-aware prioritization layer beyond disease-level repurposing alone. Second, the literature module systematically aggregates gene-drug mention evidence from PubMed to surface associations that may not yet be represented in curated expert databases. Third, the consolidated patient-level report brings curated, graph-based, literature-derived, and trial-linked evidence into a single interpretable output that can support translational review and downstream filtering.

Despite these strengths, several limitations remain. The literature module relies on article retrieval and co-mention-based evidence surfacing, which does not by itself establish causality, treatment sensitivity, or clinical relevance. In the current implementation, the PubMed output is reduced to gene-drug mention counts and does not preserve article-level identifiers in the final merged table. In addition, the graph-based module uses structured relationships in a TxGNN-derived knowledge graph, but does not perform variant-specific TxGNN model inference. The final rankings also depend on heuristic score construction and evidence weighting. Although the revised scale-aware score improved external benchmark alignment relative to the original raw-score scheme, only 2/41 eligible samples contained a CIViC-supported therapy at rank 1 and 9/41 within the top 5, indicating that the prioritization remains imperfect and should not be interpreted as clinically calibrated. The outputs should therefore be interpreted as prioritized hypotheses for expert review rather than clinically validated recommendations.

A supplementary sensitivity analysis across seven weight configurations showed that the default weights achieved the best balance of multi-source convergence and trial linkage among top-ranked candidates, but top-1 drug identity varied for 33/50 samples under at least one alternative configuration, confirming that the ranking is not fully robust to arbitrary weight changes and should be interpreted accordingly.

Overall, IDAP provides a practical framework for integrating heterogeneous evidence streams into a single report for precision oncology interpretation. By combining curated annotations, graph-linked prioritization, PubMed-derived mention evidence, and trial metadata, the framework improves visibility into potentially relevant therapeutic hypotheses for variants and cancer contexts that remain incompletely represented in expert-curated resources. Future work should focus on improving specificity of literature-derived evidence, expanding external benchmarking, evaluating score robustness, and incorporating expert or clinical validation of top-ranked candidates.

## Supplementary Material

btag300_Supplementary_Data

## Data Availability

CPTAC Genome data of BRCA, COAD, GBM, PDAC were downloaded from the GDC data portal (https://portal.gdc.cancer.gov). NSCLC data used in this study were generated and curated by the authors of the previously published work (Song et al. 2024).

## Code availability

IDAP pipeline is freely available at https://github.com/joonan-lab/IDAP-pipeline, with full documentation at https://joonan-lab.github.io/IDAP-pipeline. An archived snapshot of the code used in this study is deposited on Zenodo (DOI: https://doi.org/10.5281/zenodo.19301367).
