# Supplementary material for: IDAP: an integrated literature- and knowledge-graph-driven evidence prioritization pipeline for precision oncology
Source: Bioinformatics. 2026 May 9;42(5):btag300. doi: 10.1093/bioinformatics/btag300 (PMC13197120; doi:10.1093/bioinformatics/btag300)
Supplement: btag300_Supplementary_Data [file btag300_supplementary_data.docx]

**Supplementary Materials**

**Table S1. Summary of IDAP performance metrics evaluated across 50 samples.**

| **Patient ID** | **Cancer** | **Variants** | **Runtime (s)** | **API Calls** | **Memory (MB)** | **Total Drugs** | **Mean Score** | **Max Score** |
| --- | --- | --- | --- | --- | --- | --- | --- | --- |
| 01CO022 | COAD | 3061 | 1112.5 | 2945 | 809.1 | 2562 | 1.3 | 58.4 |
| 01CO014 | COAD | 1467 | 628.9 | 1440 | 792.5 | 1201 | 1.2 | 31.2 |
| C3L-00677 | GBM | 1873 | 682.5 | 1322 | 771.6 | 1073 | 1.2 | 34.4 |
| RE-P006-T | NSCLC | 548 | 524.8 | 1244 | 985.9 | 976 | 1.0 | 12.0 |
| RE-P001-T | NSCLC | 593 | 509.2 | 1142 | 1697.7 | 907 | 1.6 | 20.1 |
| RE-P007-T | NSCLC | 529 | 484.5 | 1115 | 861.2 | 878 | 1.5 | 15.5 |
| 01BR018 | BRCA | 343 | 329.6 | 609 | 952.0 | 473 | 1.6 | 16.7 |
| RE-P008-T | NSCLC | 271 | 333.6 | 599 | 868.6 | 430 | 1.5 | 14.7 |
| 05CO002 | COAD | 137 | 216.6 | 432 | 919.0 | 325 | 1.7 | 9.6 |
| 01CO015 | COAD | 149 | 216.3 | 325 | 801.6 | 234 | 2.4 | 9.9 |
| 01CO019 | COAD | 96 | 195.6 | 309 | 803.2 | 225 | 2.3 | 10.4 |
| RE-P002-T | NSCLC | 121 | 243.0 | 322 | 950.2 | 219 | 2.4 | 10.2 |
| 01CO008 | COAD | 192 | 217.9 | 288 | 793.8 | 205 | 2.4 | 10.4 |
| 01BR010 | BRCA | 52 | 225.6 | 304 | 1262.6 | 201 | 2.3 | 12.8 |
| C3L-01048 | GBM | 118 | 179.0 | 287 | 1224.5 | 195 | 2.6 | 9.1 |
| 01BR020 | BRCA | 104 | 203.1 | 254 | 818.0 | 181 | 2.5 | 8.8 |
| C3L-00277 | PDAC | 41 | 155.1 | 227 | 906.9 | 165 | 2.8 | 8.8 |
| C3L-01045 | GBM | 88 | 170.2 | 240 | 1230.4 | 161 | 2.4 | 10.4 |
| C3L-00674 | GBM | 53 | 153.1 | 215 | 1428.2 | 150 | 3.1 | 10.4 |
| C3L-00589 | PDAC | 115 | 169.2 | 187 | 907.0 | 136 | 3.2 | 8.8 |
| 01CO005 | COAD | 165 | 197.9 | 183 | 904.2 | 131 | 3.3 | 8.8 |
| RE-P005-T | NSCLC | 182 | 206.6 | 181 | 1434.1 | 128 | 3.6 | 13.6 |
| C3L-01046 | GBM | 59 | 143.4 | 166 | 1223.6 | 124 | 3.5 | 8.8 |
| C3L-00365 | GBM | 98 | 158.3 | 164 | 907.3 | 110 | 4.1 | 9.6 |
| 01BR008 | BRCA | 88 | 182.5 | 148 | 1235.8 | 109 | 4.3 | 10.1 |
| RE-P009-T | NSCLC | 49 | 166.8 | 139 | 903.0 | 102 | 2.3 | 8.8 |
| C3L-00017 | PDAC | 91 | 158.6 | 138 | 912.8 | 100 | 4.4 | 9.6 |
| C3L-01043 | GBM | 48 | 132.4 | 129 | 784.5 | 89 | 4.0 | 8.8 |
| C3L-01049 | GBM | 56 | 124.5 | 124 | 1222.2 | 89 | 2.7 | 9.6 |
| C3L-00401 | PDAC | 19 | 144.9 | 130 | 788.1 | 87 | 3.5 | 8.9 |
| C3L-00622 | PDAC | 44 | 135.2 | 115 | 1216.0 | 87 | 3.9 | 9.5 |
| C3L-00625 | PDAC | 37 | 141.2 | 118 | 894.8 | 87 | 3.8 | 8.8 |
| C3L-00102 | PDAC | 29 | 131.0 | 95 | 862.7 | 76 | 4.2 | 8.0 |
| RE-P010-T | NSCLC | 25 | 130.7 | 97 | 920.1 | 75 | 3.5 | 8.0 |
| C3L-01040 | GBM | 119 | 138.8 | 96 | 906.4 | 74 | 5.6 | 8.8 |
| 01CO013 | COAD | 90 | 140.7 | 102 | 786.6 | 69 | 3.3 | 8.8 |
| RE-P004-T | NSCLC | 106 | 148.8 | 81 | 782.6 | 67 | 5.0 | 8.8 |
| 01CO001 | COAD | 92 | 136.7 | 80 | 893.2 | 66 | 6.2 | 9.6 |
| 01BR015 | BRCA | 61 | 146.6 | 94 | 1224.4 | 64 | 2.8 | 8.0 |
| 01BR023 | BRCA | 22 | 122.2 | 76 | 984.3 | 62 | 6.5 | 8.9 |
| C3L-00104 | GBM | 89 | 120.7 | 54 | 848.8 | 55 | 7.4 | 8.8 |
| 01BR001 | BRCA | 68 | 129.6 | 62 | 788.8 | 55 | 7.3 | 8.8 |
| C3L-00599 | PDAC | 58 | 104.3 | 49 | 1215.7 | 54 | 6.0 | 8.0 |
| C3L-00598 | PDAC | 60 | 113.6 | 51 | 1212.3 | 50 | 8.2 | 8.8 |
| RE-P003-T | NSCLC | 7 | 91.8 | 16 | 901.1 | 38 | 2.2 | 4.0 |
| C3L-00189 | PDAC | 21 | 98.8 | 35 | 963.2 | 35 | 3.9 | 8.0 |
| 01BR009 | BRCA | 29 | 107.5 | 44 | 785.7 | 30 | 3.1 | 8.3 |
| 01BR017 | BRCA | 20 | 97.8 | 30 | 969.6 | 17 | 1.0 | 8.0 |
| 01CO006 | COAD | 7 | 87.9 | 18 | 887.4 | 11 | 0.3 | 0.6 |
| 01BR025 | BRCA | 1 | 67.8 | 1 | 894.2 | 2 | 2.0 | 2.0 |

Each row represents one patient sample. Columns include cancer type, variant counts, per-module runtime (seconds), API call counts, peak memory usage (MB), drug counts by module, TxGNN drug category distribution, evidence source overlap counts, and combined score statistics. BRCA, breast cancer; COAD, colon adenocarcinoma; GBM, glioblastoma; NSCLC, non-small cell lung cancer; PDAC, pancreatic adenocarcinoma. *Note: The full table with all 31 columns (per-module runtimes, per-module drug counts, TxGNN category distributions, source overlap counts) is provided in the accompanying Excel file.*

**Table S2. Sensitivity analysis of combined score weights across seven configurations.**

| **Configuration** | **w_graph** | **w_lit** | **w_curated** | **Top-1 multi-source (%)** | **Top-1 with trial (%)** | **Top-5 multi-source (mean)** | **Top-5 with trial (mean)** | **Median tau vs Default** |
| --- | --- | --- | --- | --- | --- | --- | --- | --- |
| Default (0.50/0.40/0.10) | 0.5 | 0.4 | 0.1 | 62 | 92 | 2.3 | 4.3 | — |
| Equal (0.33/0.33/0.33) | 0.3 | 0.3 | 0.3 | 62 | 78 | 2.3 | 3.9 | 0.439 |
| Graph-heavy (0.70/0.20/0.10) | 0.7 | 0.2 | 0.1 | 62 | 88 | 2.3 | 4.0 | 0.427 |
| Lit-heavy (0.20/0.70/0.10) | 0.2 | 0.7 | 0.1 | 60 | 94 | 1.7 | 4.5 | 0.205 |
| Curated-heavy (0.20/0.20/0.60) | 0.2 | 0.2 | 0.6 | 32 | 52 | 1.5 | 3.2 | 0.414 |
| Graph+Curated (0.45/0.10/0.45) | 0.4 | 0.1 | 0.4 | 52 | 70 | 2.0 | 3.4 | 0.427 |
| Lit+Curated (0.10/0.45/0.45) | 0.1 | 0.4 | 0.4 | 56 | 76 | 1.9 | 3.7 | 0.21 |

Seven weight configurations were tested for the percentile-normalized combined score. For each configuration, the three percentile weights (w_graph, w_literature, w_curated) were varied while holding convergence (0.20), curated-bonus (0.30), and trial-flag (0.05) coefficients fixed. Top-1 multi-source (%) indicates the percentage of samples whose top-ranked candidate was supported by at least two evidence sources. Top-1 with trial (%) indicates the percentage with associated ClinicalTrials.gov metadata. Top-5 multi-source mean is the average number of top-5 candidates per sample supported by two or more evidence layers. The default configuration (0.50/0.40/0.10, bold) achieved the highest balance of multi-source convergence and trial linkage. Kendall tau rank correlation between the default and each alternative configuration is also shown; median tau values ranged from 0.21 to 0.44, indicating moderate rank agreement across weight choices.


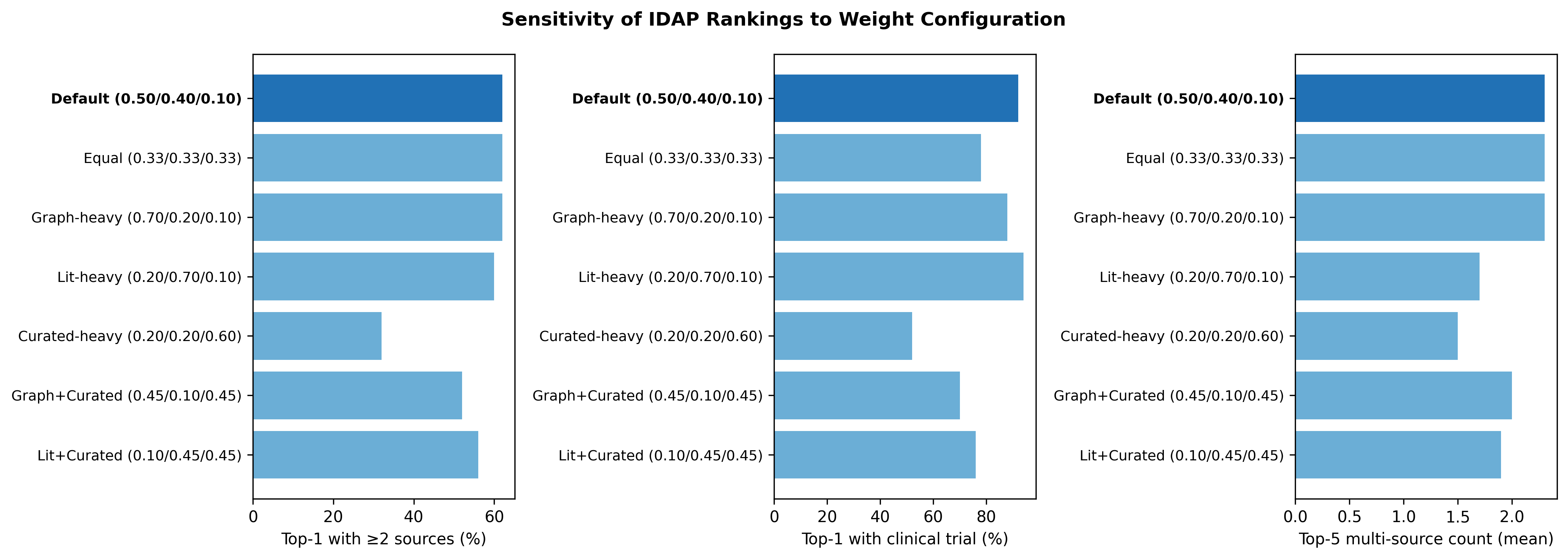


**Supplementary Figure S1. Sensitivity of IDAP rankings to weight configuration.** Bar charts showing three key ranking quality metrics across seven weight configurations for the percentile-normalized combined score. (Left) Percentage of samples whose top-ranked candidate was supported by at least two evidence sources. (Center) Percentage of samples whose top-ranked candidate had associated ClinicalTrials.gov metadata. (Right) Mean number of top-5 candidates per sample with multi-source support (≥2 evidence layers). The default configuration (0.50/0.40/0.10, dark blue) achieved the highest proportion of top-ranked candidates with multi-source support (62%) and clinical trial linkage (92%). Top-1 drug identity was stable across all seven scenarios for 17/50 samples, with a mean of 1.98 distinct top-1 drugs per sample, indicating moderate sensitivity to weight choice but reasonable robustness for the highest-ranked outputs.
